# Effect of Deep Brain Stimulation on Cerebellar Tremor Compared to Non-Cerebellar Tremor Using a Wearable Device in a Patient With Multiple Sclerosis: Case Report

**DOI:** 10.3389/fnhum.2021.754091

**Published:** 2022-01-13

**Authors:** Tao Xie, Mahesh Padmanaban, Adil Javed, David Satzer, Theresa E. Towle, Peter Warnke, Vernon L. Towle

**Affiliations:** ^1^Department of Neurology, University of Chicago Medicine, Chicago, IL, United States; ^2^Department of Neurosurgery, University of Chicago Medicine, Chicago, IL, United States

**Keywords:** DBS, cerebellar tremor, accelerometer, multiple sclerosis, case report

## Abstract

Tremor of the upper extremity is a significant cause of disability in some patients with multiple sclerosis (MS). The MS tremor is complex because it contains an ataxic intentional tremor component due to the involvement of the cerebellum and cerebellar outflow pathways by MS plaques, which makes the MS tremor, in general, less responsive to medications or deep brain stimulation (DBS) than those associated with essential tremor or Parkinson's disease. The cerebellar component has been thought to be the main reason for making DBS less effective, although it is not clear whether it is due to the lack of suppression of the ataxic tremor by DBS or else. The goal of this study was to clarify the effect of DBS on cerebellar tremor compared to non-cerebellar tremor in a patient with MS. By wearing an accelerometer on the index finger of each hand, we were able to quantitatively characterize kinetic tremor by frequency and amplitude, with cerebellar ataxia component on one hand and that without cerebellar component on the other hand, at the beginning and end of the hand movement approaching a target at DBS Off and On status. We found that cerebellar tremor surprisingly had as good a response to DBS as the tremor without a cerebellar component, but the function control on cerebellar tremor was not as good due to its distal oscillation, which made the amplitude of tremor increasingly greater as it approached the target. This explains why cerebellar tremor or MS tremor with cerebellar component has a poor functional transformation even with a good percentage of tremor control. This case study provides a better understanding of the effect of DBS on cerebellar tremor and MS tremor by using a wearable device, which could help future studies improve patient selection and outcome prediction for DBS treatment of this disabling tremor.

## Introduction

Tremor of the upper extremity is one of the major causes of disability in multiple sclerosis (MS). Although the exact prevalence is unknown, one study found that 58% of participants had tremor, and of these, 27% had a tremor-related disability, and 10% had incapacitating tremor (Alusi et al., 2001). MS tremor is complex, in that it contains a postural and kinetic tremor as in essential tremor (ET) and an additional ataxic intentional tremor component of cerebellar dysfunction in many cases (Koch et al., 2007), which is likely due to the involvement of the cerebellum and cerebellar outflow pathways by MS plaques (Alusi et al., 2001). MS tremor is often poorly responsive to medications commonly used for ET, such as primidone and propranolol (Roy and Aziz, 2014). The effect of deep brain stimulation (DBS) of a ventral intermediate nucleus (VIM) on the control of hand tremor in patients with MS is highly variable and, in general, the tremor is less responsive than those associated with ET or Parkinson's disease (PD; Roy and Aziz, 2014), even in DBS targeting posterior subthalamic area (PSA) or caudal zona incerta (cZi; Xie et al., 2012a; Ramirez-Zamora et al., 2016), or combined targets (Oliveria et al., 2017). Even in those with improved tremor following surgery, the improvement does not always translate to improved functional status (Roy and Aziz, 2014).

We hypothesized that patients with MS with a tremor of the cerebellar ataxic component could still have a good response to DBS in tremor suppression as in rhythmic postural and action tremor of ET type, except that the distal oscillation would prevent transformation into a functional benefit. We would test this hypothesis through the use of a wearable accelerometer on the index finger approaching a target with a subsequent waveform analysis of amplitude and frequency to quantitatively characterize the different tremors in each individual hand at the beginning and end of the target approaching movement at DBS Off and On status. The hand function is also captured in the Supplementary Video Clips. This study could help decipher the effect of DBS on cerebellar tremor and function control and help future studies to improve patient selection and outcome prediction in patients with MS undergoing DBS.

## Methods

### Case Description

The subject was an 18-year-old man with a 7-year history of relapsing-remitting MS and stable MS symptoms for more than 1 year on Natalizumab 300 mg IV every month for ≥6 months when he visited us in 2015. He had severe tremor on the right hand, with prominent postural tremor and kinetic tremor for more than 3 years, which had a significant cerebellar component of distal oscillation. He also had moderate postural and kinetic tremor on the left hand without significant distal cerebellar oscillation. He virtually lost hand function (worse on the right than the left) for at least 1 year prior to his visit to us due to the gradual worsening of his tremor. He had severe truncal ataxia as well for which he had been wheelchair-bound for years. His hand tremor failed to respond to primidone 125 mg po tid and propranolol 30 mg po tid. He had no MS plaques in VIM and PSA/cZi areas and no evidence of enhancing lesions in brain MRI with contrast. A decision was made to implant bilateral VIM/cZi DBS (Medtronic 3387, MN, USA) by our team to improve his hand tremor.

Prior to the DBS procedure, a CT and MRI of the brain were fused, and the Schaltenbrand atlas was superimposed over the imaging to help plan the trajectory to the anatomical target of VIM/cZi. The intraoperative microelectrode recordings were used to define the electrophysiological target. The macrostimulation test was applied to assess the clinical effectiveness and adverse effect profile and further refined the target with minor intraoperative adjustments of the DBS leads. Fusion of MRI with intraoperative CT demonstrated that macroelectrode tips located at the border of VIM and cZi, with stereotactic coordinates of (−12.4, −4.8, +1.6) on the left and (+11.9, −4.9, +2.7) on the right (medial-lateral, anterior-posterior, and superior-inferior coordinates, respectively, given in mm relative to the midcommissural point and midsagittal plane). The ventral contacts yielded best tremor suppression, with the settings of C+/0–, amplitude 4.2 V, pulse width 60 μs, and frequency 180 Hz for the left lead, and C+/8–, amplitude 3.8 V, pulse width 60 μs, and frequency 180 Hz for the right lead at 1 year after the DBS placement.

The finger trajectories were captured as follows: Motion was encoded by an accelerometer embedded in a cardboard tube (5 cm long, 2 cm diameter), which was placed over the index finger of the subject. A small shielded cable was dressed up the arm to the shoulder, allowing unencumbered movement. The three X, Y, and Z channels were fed into electroencephalogram amplifiers (0.1–50 Hz) and averaged, yielding a single movement trajectory over time. The quantitative amplitude and frequency were automatically transformed into conventional power spectrums.

The Fahn-Tolosa-Marin Tremor Rating Scale (FTMTRS) was used to assess the overall tremor score, right upper extremity tremor score (rest, posture/intention), left upper extremity tremor score, right hand function score (in drawing A, B, C, and pouring water) and left hand function score at DBS On state (for 15 min) compared to DBS Off (for 15 min) state. The Scale for the Assessment and Rating of Ataxia (SARA) was also used to assess the overall ataxia score, right upper extremity ataxia score (finger chase, nose-finger test, and fast alternating hand movements), and left upper extremity ataxia score at DBS On state compared to DBS Off state.

**Figure 1:**
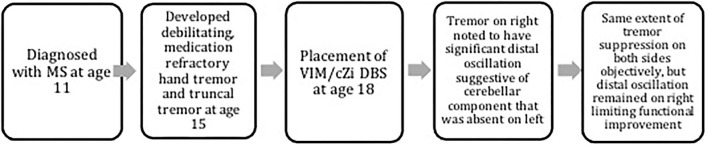
The timeline of events. The timeline of the events is depicted in the flowchart.

**Figure 2:**
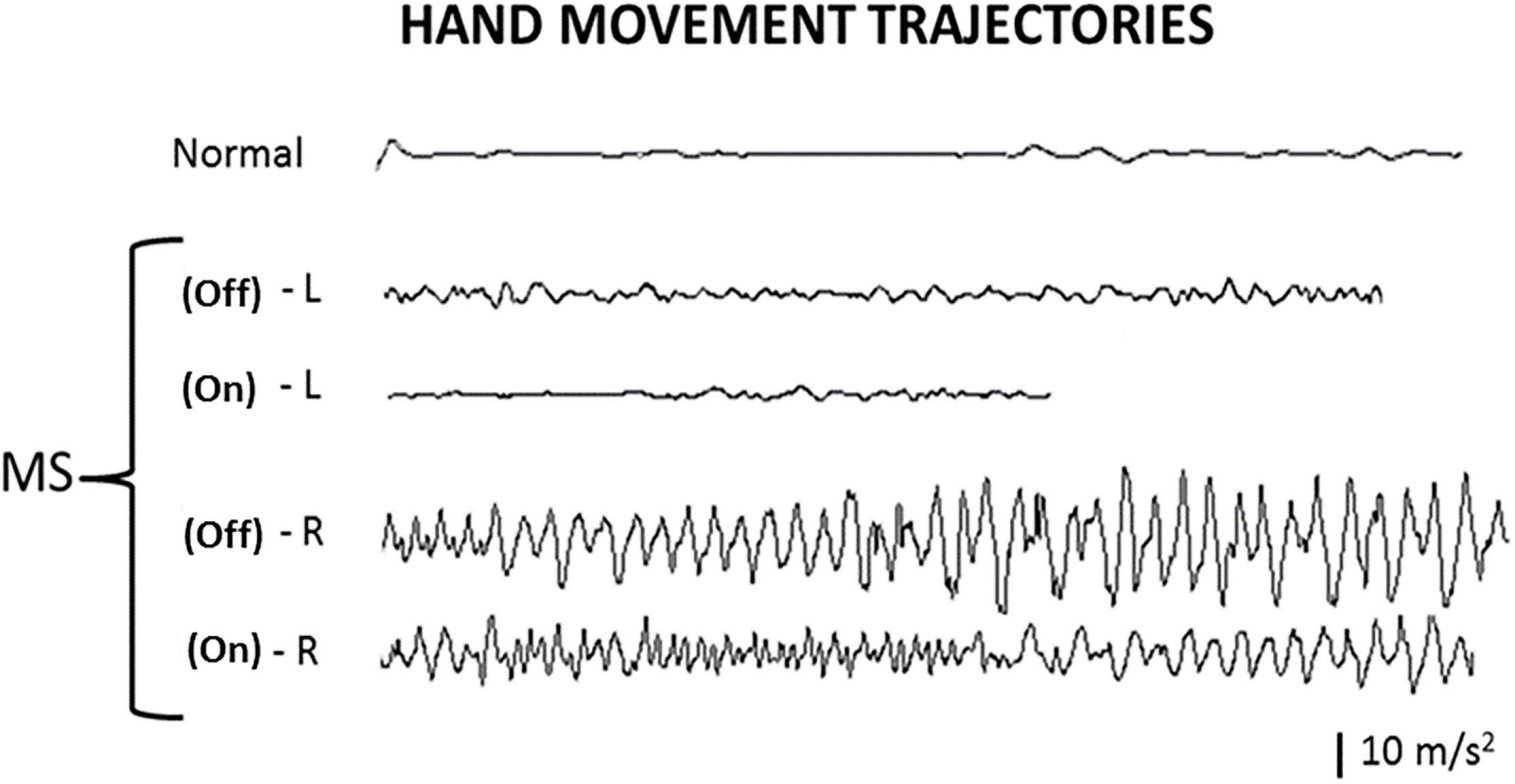
Hand movement trajectories. Index finger movement trajectories in the finger-to-finger task illustrate the tremor amplitudes and frequencies of the hands, with deep brain stimulation (DBS) Off and On. Normal, normal healthy person; L, left hand; R, right hand; Off, DBS Off; On, DBS On.

**Figure 3:**
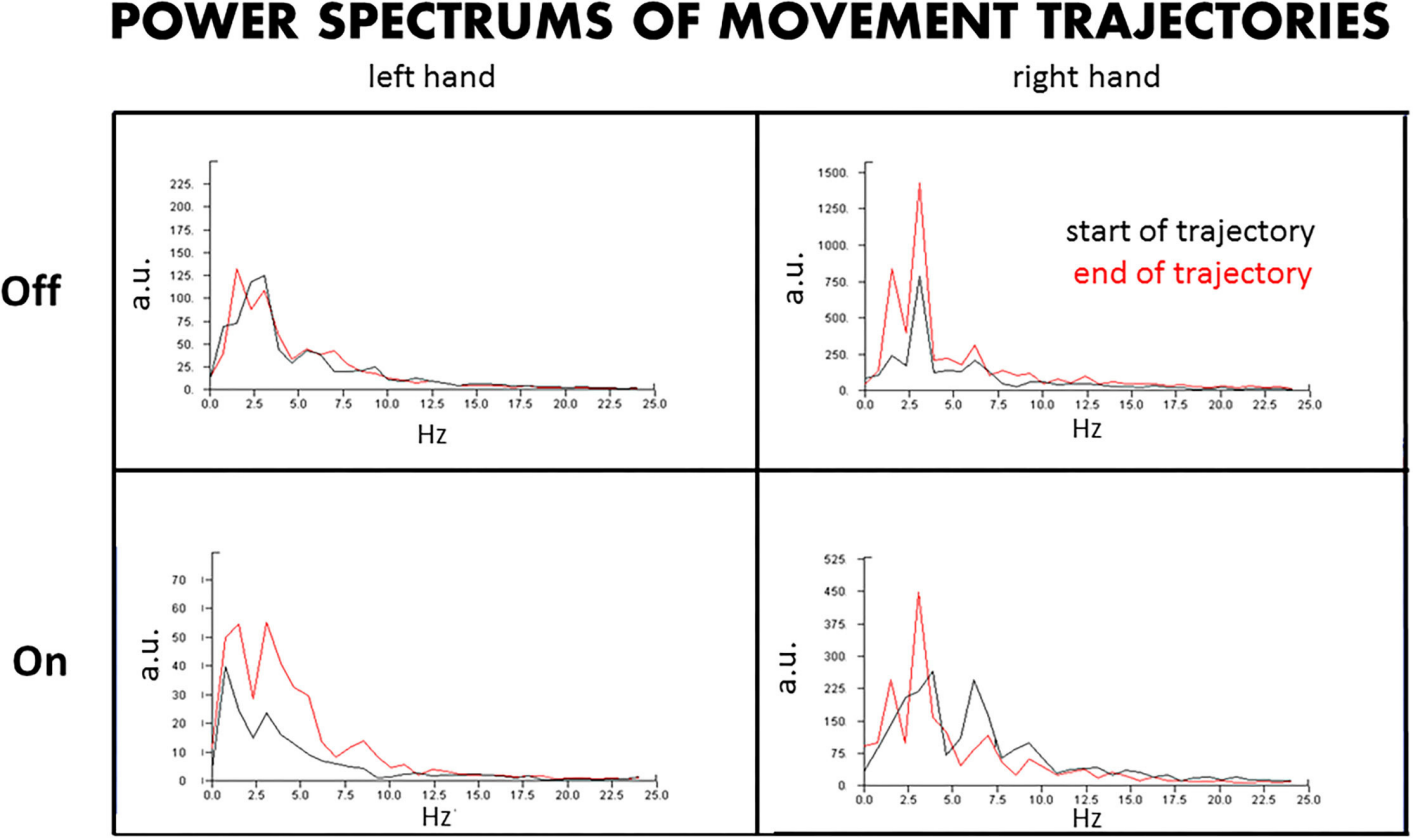
Power spectrum of the hand trajectories. The power spectrum of the hand trajectories reveals the amplitudes and dominant frequencies of the tremors at the beginning and end of the movements approaching the target, with DBS Off and On. L, left hand; R, right hand; Off, DBS Off; On, DBS On; a.u., arbitrary units.

The timeline of events is demonstrated in Figure 1. Finger trajectories are displayed in Figure 2, and the quantitative amplitude and frequency are displayed in Figure 3.

## Results

### Hand Movement Trajectories With DBS Off and On

By visual inspection (Figure 2), the left hand in a non-MS non-DBS normal person as a control and the left hand in the patient with MS had no significant change in waveform (amplitude and frequency pattern) at the end of the test (when the finger of the patient was getting close to the finger of the examiner, <3 cm away) compared to that at the beginning of the test (when the patient was holding up a finger 30 cm away from the finger of the examiner), during both the DBS Off and DBS On states. In contrast, the right hand in the patient with MS had a significant change in the waveform at the end of the trajectory or movement compared to that at the beginning of the trajectory, during both the DBS Off and DBS On states. Notably, the overall tremor amplitudes were reduced on both hands at the DBS On state compared to the DBS Off state.

### Power Spectrum of the Hand Trajectories With DBS Off and On

By the amplitude and frequency analysis (Figure 3), the left hand had no significant change in amplitude and frequency at the end of the test compared to that at the beginning of the test, at both DBS Off and On states (Figure 3, left upper panel), although the amplitude was reduced by about 60% at DBS On state (Figure 3, left lower panel). In contrast, the right hand had a significant change in amplitude and frequency at the end of the trajectories compared to that at the beginning, with doubled amplitude and doubled peaks of different frequencies at the end of the test, at both DBS Off (Figure 3, right upper panel) and On states (Figure 3, right lower panel), although the amplitude was reduced by about 60% at DBS On state.

### Hand Function With DBS Off and On

Although the tremor is improved on both hands after the DBS surgery (DBS On compared to DBS Off), a better functional improvement is observed on the left hand compared to the right hand when the hand extended to the distal targets with DBS On (Supplementary Video Clips). FTMTRS for the overall tremor score was improved by 40%; right upper extremity tremor score (rest, posture/intention) was improved by only 37%, but left upper extremity tremor score improved by 66%; the right hand function score (in drawing A, B, C, and pouring water) improved by only 25%, but the left hand function score improved by 50%, all at DBS On state compared to DBS Off state. SARA for the overall ataxia score was improved by 23%; the right upper extremity ataxia score (finger chase, nose-finger test, and fast alternating hand movements) improved by only 33%, but the left upper extremity ataxia score improved by 62% at DBS Off state compared to DBS On state.

## Discussion

In the patient with MS, undergoing bilateral VIM/cZi DBS, reduction in tremor amplitude was similar regardless of the absence or presence of cerebellar-type tremor, but the functional benefit was limited by the presence of cerebellar-type tremor. The cerebellar ataxia component was observed in the right hand, with the distal oscillation of reduced frequency and increased amplitude when the hand approached the target. Although the suppression of the tremor by DBS was similar (by 60%) at both the beginning and the end of the finger-to-nose test on both types of tremors as captured by the accelerometer, the hand function was significantly different, with much worse function on the right hand with cerebellar ataxia compared to the left hand without significant cerebellar ataxia component, as shown by the Supplementary Video Clips and the FTMTRS and SARA scores on distal hand function, as the distal amplitude of the right hand was much worse due to the distal oscillation of the ataxia. This could also explain why the patient with MS often holds the hand close to the trunk when they use the ataxic hand, as it could reduce the distal oscillation and make the hand steadier with a better function. It is interesting to know that in a relatively well-controlled study with detailed analysis, in this study, we found that cerebellar tremor in fact could be suppressed by DBS. It is the oscillation when approaching the target that increased the amplitude (by about 2-fold) in ataxia that makes the hand function poorly controlled compared to that without significant distal oscillation. It also suggests that ataxia is not necessarily an absolute contraindication for DBS in carefully selected cases with less distal amplitude by oscillation, as the greater amplitude would impair the hand function otherwise. Tremor with ataxia component could have a reasonable response to DBS, as reported in a patient with fragile X-associated tremor/ataxia syndrome (Xie et al., 2012b), and two other case reports as well (Cury et al., 2019; Barcelos et al., 2020), although in this study we wanted to explore more on why a nice tremor suppression by DBS is unable to be transformed to functional gain and how we can predict the responsiveness of the cerebellar tremor to DBS (such as how big the distal oscillation in cerebellar tremor could affect the function gain), which could help proper selection of DBS candidates with cerebellar tremor and would have a more broad clinical application. The modern accelerometer could easily adapt a new program to assess the ataxia and oscillation by automatically comparing the distal to the proximal amplitude and frequency, and possibly even be able to do a three-dimensional comprehensive analysis as well. Given the overall limited functional control of MS tremor by DBS even in well-selected targets (Xie et al., 2012a; Ramirez-Zamora et al., 2016; Oliveria et al., 2017), the proof of the concept as demonstrated in this study by this limited case report should be further validated by a clinical trial or serial cases on MS tremor for the better selection of patients with MS and the better prediction of their outcome for DBS.

### Patient Perspective

The patient provided a written consent for this study and publication. He has been happy with the improved tremor control and hand function, particularly on his left hand. His hand tremor and function remain stable as described during the 5-year follow-up, without side effects being reported so far.

## Data Availability Statement

The original contributions presented in the study are included in the article/Supplementary Material, further inquiries can be directed to the corresponding author/s.

## Ethics Statement

Ethical review and approval was not required for the study on human participants in accordance with the local legislation and institutional requirements. The patients/participants provided their written informed consent to participate in this study. Written informed consent was obtained from the individual(s) for the publication of any potentially identifiable images or data included in this article.

## Author Contributions

TX contributed to conception, design, and drafting the article. TX, MP, AJ, DS, and VT contributed to data collection, analysis, and interpretation. All authors contributed to critical revision of the article and have read and approved the final version of the article.

## Conflict of Interest

The authors declare that the research was conducted in the absence of any commercial or financial relationships that could be construed as a potential conflict of interest.

## Publisher's Note

All claims expressed in this article are solely those of the authors and do not necessarily represent those of their affiliated organizations, or those of the publisher, the editors and the reviewers. Any product that may be evaluated in this article, or claim that may be made by its manufacturer, is not guaranteed or endorsed by the publisher.

## Supplementary Material

Supplementary Video Clip 1 DBS Off.

Supplementary Video Clip 2 DBS On. Hand function at DBS Off and On. Although the tremor is improved on both hands after the DBS surgery (DBS On in Clip 2 compared to DBS Off in Clip 1), a better function is observed on the left hand compared to the right hand when the hand extended to the distal targets with DBS On (Clip 2).

## Abbreviations

DBS: deep brain stimulation
MS: multiple sclerosis
VIM: ventral intermediate nucleus
PSA: posterior subthalamic area
cZi: caudal zona incerta.
